# High-throughput sequencing revealed that microRNAs were involved in the development of superior and inferior grains in bread wheat

**DOI:** 10.1038/s41598-018-31870-z

**Published:** 2018-09-14

**Authors:** Yongyan Wang, Chaonan Shi, Tianxiao Yang, Lei Zhao, Jianhui Chen, Ning Zhang, Yan Ren, Guiliang Tang, Dangqun Cui, Feng Chen

**Affiliations:** 1 Agronomy College/National Key Laboratory of Wheat and Maize Crop Science/Collaborative Innovation Center of Henan Grain Crops, Henan Agricultural University, 15 Longzihu College District, Zhengzhou, 450046 China; 2 Department of Biological Sciences, Life Science and Technology Instituted, Michigan Technological University, Houghton, MI 49931 USA

## Abstract

High-throughput sequencing was employed to investigate the expression of miRNAs and their target genes in superior and inferior seeds of Aikang 58. Small RNA sequencing revealed 620 conserved and 64 novel miRNAs in superior grains, and 623 conserved and 66 novel miRNAs in inferior grains. Among these, 97 known miRNAs, and eight novel miRNAs showed differential expression between the superior and inferior seeds. Degradome sequencing revealed at least 140 candidate target genes associated with 35 miRNA families during the development of superior and inferior seeds. GO and KEGG pathway analysis showed that the differentially expressed miRNAs, both conserved and novel, were likely involved in hormone production, carbohydrate metabolic pathways, and cell division. We validated eight known and four novel grain development-related miRNAs and their target genes by quantitative real-time polymerase chain reaction to ensure the reliability of small RNA and degradome-seq results. Of these, miR160 and miR165/166 were knocked down in Arabidopsis using short-tandem target mimic (STTM160 and STTM165/166) technology, which confirmed their roles in seed development. Specifically, STTM160 showed significantly smaller grain size, lower grain weight, shorter siliques length, shorter plant height, and more serrated leaves, whereas STTM165/166 showed decreased seed number, disabled siliques, and curled upward leaves.

## Introduction

MicroRNAs (miRNAs) are a class of short 20–24 nucleotide (nt) sRNAs produced from endogenous non-coding and imperfectly complementary (stem-loop) RNA precursors that can be transcribed into single-stranded primary miRNAs by RNA polymerase II^1,2^. MicroRNAs regulate gene expression by sequence-specific base pairing with messages of protein-coding target genes^3,4^. Studies of plant miRNAs have indicated that most groups of these miRNAs played crucial roles in development, biological function, responses to environmental signals and maintenance of tissues and cells^5^. Although extensive studies of miRNAs have been conducted in model plants, relatively few have focused on small RNAs in wheat because of incomplete reference sequences. Therefore, high-throughput sequencing strategies have been employed to better understand miRNAs and identify new miRNAs in cereal crops, such as rice, maize, and wheat. For example, Meng *et al*. used high-throughput sequencing and genome-wide mining to identify 104 miRNAs which were potentially involved in the regulation of grain development^6^. Li *et al*. identified a total of 186 known miRNAs and 37 novel miRNAs during grain development using a combination of small RNA and degradome sequencing techniques^7^.

As one of the world’s most important staple foods, bread wheat (*Triticum aestivum* L.) is essential to food security and provides approximately 20% of human dietary calories^8^. Grain filling is a key transitional process during seed production. During grain filling, differences in grain size develop in different positions of the same spike, resulting in superior and inferior grains. Currently, increasing the weight of inferior seeds is one of the primary ways to enhance wheat yield. Recently, an increasing number of miRNAs related to grain size and grain development have been identified in crops. In wheat, miR156-regulated SBP-box (SQUAMOSA promoter binding protein–like) function antagonistically interact with *DWARF53* to regulate *TEOSINTE BRANCHED1* and *BARRE STALK1* expression during bread wheat tillering and spikelet development^9^. Activity of miR172, which plays a key role in wheat spike morphology and grain threshability, was inhibited via a miRNA target mimic to generate compact spikes and transition from glumes to florets in distal spikelets^10^. MiR9678 affects seed germination by generating phased siRNAs and modulating abscisic acid/gibberellin signaling in wheat^11^. In rice, *OsSPL16* (*SQUAMOSA-PROMOTER BINDING PROTEIN-LIKE* transcription factor; i.e., *OsGW8*), the target gene of miR156, has been shown to positively regulate grain size by promoting cell division and grain filling^12^. Moreover, the target gene of miR397, *OsLAC* (*laccase*), increased grain size and promoted panicle branching to improve rice yield^13^. MiR159 positively regulates organ size, including stem, leaf, and grain size in rice^14^. To better identify the function of miRNA, the short-tandem target mimic (STTM) method was developed as a powerful technology to silence numerous miRNA family functions in plants^15^. To date, numerous miRNAs have been functionally verified via STTM in different species^16–18^. More recently, STTM was used to verify that some miRNAs (miR156, miR160, miR166, miR171, miR398, miR441, and miR1428) in rice regulate important agronomic traits^19^. For example, STTM398 lines possessed significantly reduced grain number and decreased grain length and width in rice.

Aikang 58 (AK58) is a semi-winterness bread wheat characterized by high, stable yield and resistance to cold and multiple diseases. Because of its excellent characteristics, AK58 is currently one of the most popular cultivars. Indeed, AK58 had one of the largest cumulative planting areas in China and won the first prize in Chinese national science and technology progress. However, the grain weight and uniformity of grain size of AK58 can vary greatly in the same plant. Further analysis showed that the small seeds are mainly from spikelets near the top (i.e., inferior grains) and large seeds are mainly from the spikelets near the bottom (i.e., superior grains) in spikes of AK58. In rice, differentially expressed miRNAs were involved in diverse developmental processes in superior and inferior grains during rice grain filling, and improvement of inferior grains weight was crucial to enhanced rice yield^20^. Therefore, to further increase the yield of AK58, the inferior grains weight should receive increased attention. Here, we employed small RNA sequencing and degradome sequencing to investigate miRNAs and their targets associated with development of grain size during production of superior and inferior grains in spikes of AK58. This study provided useful information to uncover miRNAs involved in development of grain size and to further understand the genetic basis of grain size in bread wheat.

## Materials and Methods

### Plant materials

**Figure 1:**
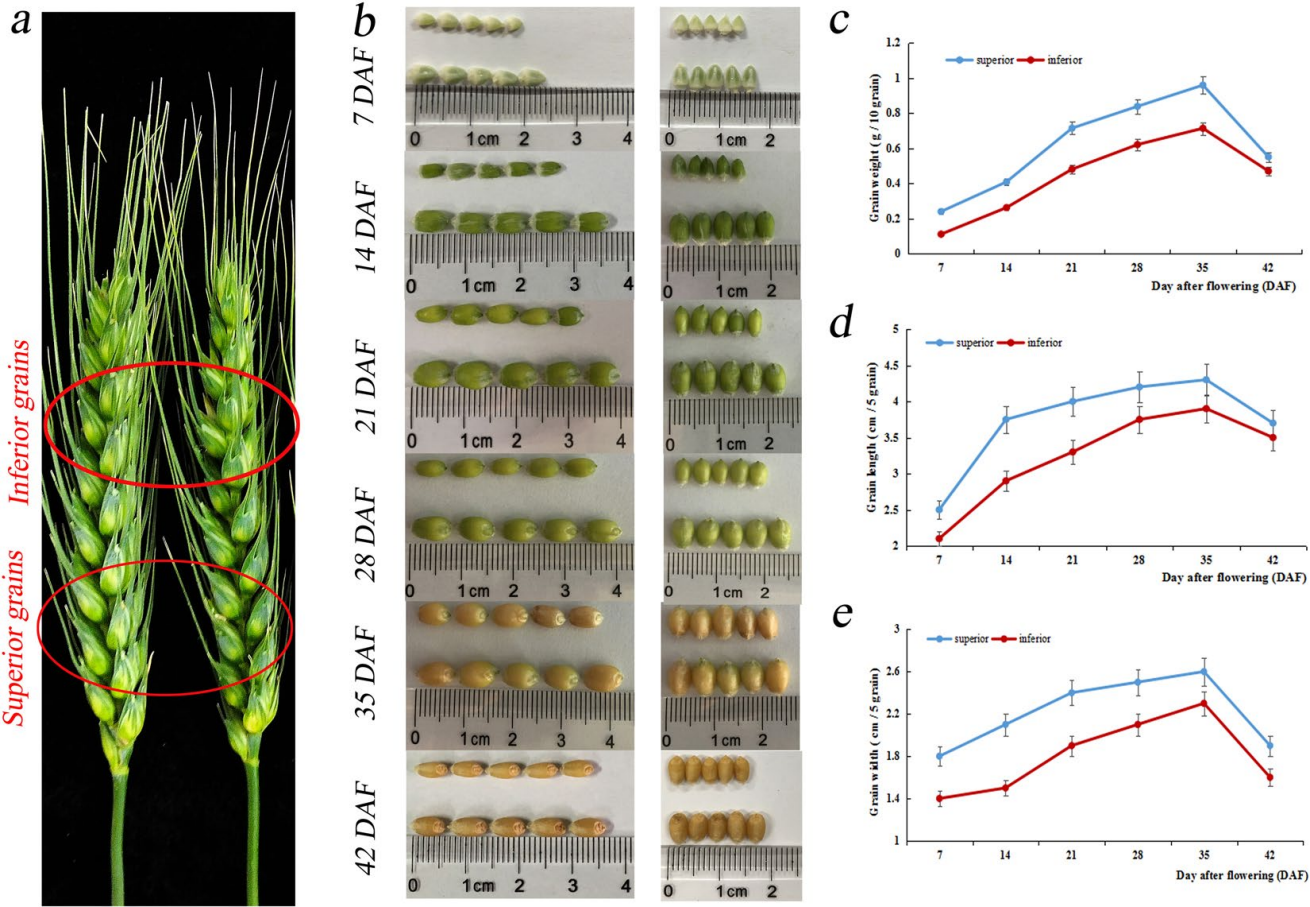
Kernel size comparison of superior and inferior grains of AK58.

A Chinese semi-winterness bread wheat cultivar, Aikang 58 (AK58, released No. Guoshenmai 2005008), was planted at the Zhengzhou Scientific Research and Education Center of Henan Agricultural University (113.6°E; 34.9°N) during the 2013–2014 planting seasons under non-stress natural and uniform soil conditions. We sampled superior grains from the six fertile spikelets on the bottom of the spike and inferior grains from the six fertile spikelets on the top of spike (Fig. 1a) at 7, 14, 21, 28, 35, and 42 days after flowering (DAF). Additionally, we harvested superior grains from the three basal rows of a spike and collected inferior grains from the three apical fertile spikelets. Each sample had three replicates. All grains were immediately frozen in liquid nitrogen and stored at −80 °C.

### Small RNA isolation, library construction, and sequencing

Total RNA was isolated from superior and inferior grains at 7, 14, 21, 28, 35, and 42 DAF, respectively, using RNA Extraction Kite GeneAnwser (Code Biotechnology Co., Ltd., Beijing, China). Next, samples were incubated with 3 µL RNase-free DNase I, 2 µL RNasin Ribonuclease Inhibitor, 9.5 µL 10 × buffer, and 80 µL diluted total RNA for 30 min at 37 °C to remove the residual DNA and RNase A.

For small RNA library construction, we mixed the same concentration and volume of RNA for each sample (7, 14, 21, 28, 35, and 42 DAF) as superior grains pools (SGP) and inferior grains pools (IGP) with three biological replicates. We then performed sequencing as follows: mixed RNA was purified with 50% polyethylene glycol to obtain a concentration of 5%. RNA molecules in the range of 18–30 nt were then enriched and purified by polyacrylamide gel electrophoresis, after which they were ligated to adapters in the 5′and 3′ terminals of the RNA. Next, the samples used as templates were subjected to reverse transcription and polymerase chain reaction (RT-PCR), after which the purified PCR products were used for Illumina sequencing by the Illumina Hiseq. 2000 platform (Oebiotech, Shanghai, China).

We also used the SGPs and IGPs with three biological replicates to construct a degradome library for sequencing. Briefly, poly (A) RNA was extracted and ligated to a 5′ RNA adapter. The double-stranded products were then digested after RT-PCR and ligated to a 3′ dsDNA adapter. Next, we used the purified libraries to finalize the sequencing, which we performed using the Illumina/Solexa Hiseq. 2000 platform (Oebiotech, Shanghai, China).

### Bioinformatics analysis of sequencing data

For small RNA sequencing data, raw reads were first filtered (including removing low-quality reads and trimming adapter sequencing), after which we obtained clean reads (18–30 nt). We then compared the common and unique reads between two samples and mapped the high quality-tags to the reference genome by Simple Object Access Protocol (SOAP) to determine their expression and distribution in the NCBI wheat EST database and DFCI Wheat Gene Index (ftp://occams.dfci.harvard.edu/pub/bio/tgi/data/Triticum_aestivum/)^21^. We subsequently used the matched tags to query the NCBI GenBank (http://www.ncbi.nlm.nih.gov/genbank/) and Rfamv.10.1 (http://rfam.xfam.org/) databases by BLAST analysis to identify miRNAs and remove rRNA, tRNA, snRNA, and snoRNA sequences. Known miRNAs were mapped to the miRBase v.18 database (http://www.mirbase.org/) for annotation, after which expression patterns in different samples were presented based on the log2-ratio and scatter plots. Additionally, we analyzed novel small RNAs by MIREAP (https://sourceforge.net/projects/mireap/) to predict the secondary structures, the DCL1 cleavage sites, and the minimum free energy of the unannotated small RNA tags, and we identified conserved precursor sequences by MIREAP^22^. Based on the hairpin structure of a pre-miRNA and the miRBase database, we also identified the corresponding miRNA star sequence^21^.

We conducted the analysis of degradome sequencing data using a method similar to that for the analysis of small RNA sequencing mentioned earlier. We generated high-quality clean reads, after which we performed standardized analysis. Notably, we identified the miRNA-mRNA pairs using PairFinder and T-plot^22,23^. Additionally, we conducted GO functional enrichment analysis of the targets using the AmiGO program (amigo.genontology.org)^24^. Finally, we carried out interaction and pathway analyses of the targets using The Kyoto Encyclopedia of Gene and Genome (KEGG) (www.genome.jp/kegg)^25^.

### Identification of novel miRNAs and prediction of miRNA targets

To identify novel miRNAs, we excluded sRNA reads that might be from known noncoding RNAs [rRNA, tRNA, small nuclear RNA (snRNA), and small nucleolar RNA (snoRNA)]. The criteria for designation as a novel miRNA were as follows: length of 20–24 nt, precursors with a signature hairpin structure and the formation of maturation achieved by Dicer, minimum free energy of precursors of less than 18 kcal/mol, a minimum support number for the maturity sequence of precursors of at least 5, and potential miRNA sequence with less than 3 nt mispairing in the sequence of the mature and perfectly matched middle sequence^21,22^.

We used the psRNATarget program (http://bioinfo3.noble.org/psRNATarget/) to predict the potential miRNA targets^26^. The cleaved target sites were categorized into five classes that relied on the abundance of degradome tags indicative of miRNA-mediated cleavage. In category 0, the reads abundance at the cleavage site was the unique maximum on the transcript; category 1 included sequences for which abundance at the cleavage site was the maximum, but not unique; category 2 included sequences for which abundance at the cleavage site was greater than for category 1, but not the maximum; category 3 included sequences for which abundance at the cleavage site was equal to or less than the median; and the remaining sequences were classified as category 4.

### Differential expression analysis

A plot of the log2-ratio and a scatter plot revealed two principles affirming differentially expressed miRNAs between superior and inferior grains. (i) The reads of the small RNA and degradome library from superior and inferior seeds were normalized by TPM (transcripts per million) using the normalization formula: normalized expression = (actual miRNA count/total count of clean reads) × 1,000,000. (ii) After normalization of the expression of all miRNAs, fold-change was calculated and then used in combination with the fold-change and P-value to generate the log2-ratio plot and scatter plot as follows:

$$\begin{array}{c}\mathrm{Fold} \mbox{-} \mathrm{change}={\rm{log2}}\,(\mathrm{normalized}\,{\rm{expression}}\,{\rm{of}}\,{\rm{miRNA}}\,{\rm{in}}\,{\rm{inferior}}\\ \,\,\mathrm{grain}/\mathrm{normalized}\,{\rm{expression}}\,{\rm{of}}\,{\rm{miRNA}}\,{\rm{in}}\,{\rm{superior}}\,\mathrm{grain})\end{array}$$

Note: If the expression was set to 0.01, the miRNA was not expressed in superior or inferior grain. If the miRNA/fold-change/was ≥1, 0.01 < P-value < 0.05 represents a difference, whereas a P-value < 0.01 reflects that the miRNA was significantly different between the superior and inferior grain.

### Transient expression assay

To verify the regulation of miR166 to its target gene *Hox9*, a double 35 S promoter of *PHB* vector was constructed miR166*-PHB* vector to over-expression miR166 in tobacco^27^. Using the plant miRNA function transient validation system, we constructed a *Hox9-GFP* subcellular localization vector, and the *Hox9-GFP* vector infect tobacco leaf with miR166-PHB. Compared with *Hox9-GFP* vector and empty *PHB* vector, *GFP* fluorescence signal was observed using laser confocal microscope (Version: zeiss lsm710).

### Quantitative real-time RT-PCR

We validated the expression patterns of eight known miRNAs (miR160a, miR164a, miR166a, miR167a, miR1579, miR1892, miR4451, and miR5653) and four novel miRNAs (miR27, miR81, miR128, and miR176) in superior and inferior seeds of different developmental stages using quantitative real-time RT-PCR (qRT-PCR). Mir-X™ miRNA qRT-PCR SYBR® Kit (Takara, Dalian, China) was used to evaluate the expression levels of miRNAs. For the targets, 1 µg of total RNA was reverse-transcribed using a PrimeScript RT reagent Kit with gDNA Eraser (Takara Bio Inc.) to generate cDNA. Real-time PCR was then conducted using SYBR Premix Ex Taq II (Takara Bio, Dalian, China) on a CFX96 Touch™ Real-Time PCR Detection System (Bio-Rad, CA, USA). All primers for the reaction were designed and synthesized by Generay Biotech (Generay, Shanghai, China) and are listed in Supplemental Table S1. We performed three technical replicates to produce average expression levels relative to the reference using the 2^−ΔΔt^ method.

### Transformation of *Arabidopsis thaliana*

STTM is a highly effective and widely applicable technology to specifically block small RNA function in *Arabidopsis thaliana*^15^. To better construct the STTM vector, pOT2, we first modified a small size (1665 bp) subcloning vector from fruit fly by introducing a 2 × 35 S promoter, a 35 S terminator, and a specific DNA fragment known as Ploy-cis (denoted pOT2-Poly-Cis). The Poly-cis was then replaced by STTM modules via multistep reactions, such as PCR amplification, products purification, SwaI digestion, and self-ligation, which resulted in pOT2-Poly-STTM. Next, we transformed self-ligated products into *E. coli* competent cells and selected positive plasmids. Subsequently, positive plasmids were ligated into pBR322 through PCR. The PCR products that made up the STTM structure and a chloramphenicol selection marker were further sub-cloned into an altered pFGC5941 binary vector via the unique PacI site. We then selected recombinant binary plasmids using chloramphenicol and kanamycin. The final constructs were verified by DNA sequencing, and the positive clones were transformed into Arabidopsis using an Agrobacterium-mediated floral dip method^28^. Transgenic plants were identified based on resistance to BASTA.

We obtained more than 20 T_1_ transgenic Arabidopsis lines, after which we analyzed the phenotypes until we obtained T_5_ homozygous transgenic plants for STTM160 and STTM165/166 in Arabidopsis. The homozygous transgenic plants were subsequently grown in long days (16 h light/8 h dark) at 22 °C. The investigated phenotypes included grain size (width, length and weight), siliques length, siliques number per plant, grain number per silique, and flowering time for transgenic plants of STTM160 and STTM165/166, as well as their wild types (Col-0). For Col-0, STTM160, and STTM165/166, we randomly selected 200 kernels to measure grain weight with three replicates and randomly selected 10 kernels to measure grain width and length at 4 × magnification (Olympus, Tokyo, Japan). We then randomly selected 3 of the 10 kernels for further observation under the microscope. All views were magnified four times and the Image-J software was employed to measure the grain width and length and siliques length for Col-0, STTM160, and STTM165/166. Finally, we used the average of 10 plants and siliques as the siliques number per plant and grain number per silique.

## Results

### Phenotype comparison of superior and inferior seeds in different developmental stages

The uniformity of kernel size of each wheat cultivar is mainly from superior grains on the bottom of spikes and inferior grains on the top of spikes (Fig. 1a). To investigate development of superior and inferior grains, we collected AK58 superior and inferior grains at 7, 14, 21, 28, 35, and 42 DAF, which covered the greatest changes in weight and size during grain development (Fig. 1b). Superior grains from AK58 showed significantly larger kernel size and higher kernel weight than inferior grains at each of the six developmental stages (Fig. 1c–e).

### Overview of high-throughput sequencing data

Based on small RNA sequencing, we obtained a total of 24,071,134 and 25,301,033 reads from the SGP and IGP after removing the adaptor and low-quality reads, respectively, and further obtained 6,507,147 and 8,199,346 unique sRNA reads from the total reads of the superior and inferior grains. Degradome sequencing indicated that a total of 25,468,373 and 19,169,019 clean reads were obtained from the SGP and IGP, and they were composed of 8,047,259 and 4,081,576 unique sRNA reads of the SGP and IGP, respectively (Table 1).

**Table 1:** Data set summary of sequencing of two small RNA and degradome libraries.

Type	Category	Superior grain		Inferior grain	
		Count	Percent (%)	Count	Percent (%)
Small RNA data	total_reads	24545794		25736061	
	high_quality	24420362	100%	25608150	100%
	3′adapter_null	8085	0.03%	8653	0.03%
	insert_null	12018	0.05%	16868	0.07%
	5′adapter_contaminants	308925	1.27%	271496	1.06%
	smaller_than_18nt	19866	0.08%	9911	0.04%
	polyA	334	0.00%	189	0.00%
	clean_reads	24071134	98.57%	25301033	98.80%
Degradome data	Total_reads	19224689	100%	25570346	100%
	Clean_reads	19169019	99.71%	25468373	99.60%
	Low_quality_value	360	0.00%	563	0.00%
	Incorrect_size	7040	0.04%	38759	0.15%
	Contain_N_base	45159	0.23%	59100	0.23%
	Adaptor	3111	0.02%	3551	0.01%

All clean reads were composed of 16 to 30 nt length small RNAs (Supplemental Fig. 1a), and 91.89% and 92.66% ranged from 20 to 24 nt in SGP and IGP based on small RNA sequencing, respectively. Furthermore, degradome sequencing showed that 99.37% and 99.71% of clean reads had 16–30 nt lengths (Supplemental Fig. 1b) and ranged from 18 to 22 nt in SGP and IGP, and the most abundant sRNAs were 20 nt and 21 nt with a percentage of 44.26% and 54.95% in SGP and 49.80% and 48.72% in IGP.

In addition, 78.5% and 69.44% of the total sRNA sequences representing 65.14% and 65.63% of unique sRNAs (Supplemental Table S2) were found to match the genome by degradome sequencing. Total sRNA from deep sequencing contained miRNA, rRNA, tRNA, snRNA, and snoRNA, most of which showed different abundances in SGP and IGP (Table 2). In the two databases of the small RNA sequencing, 72.97% of the total sRNAs (Supplemental Fig. 2a) overlapped, whereas only 14.81% of the unique sRNAs overlapped in the SGP and IGP libraries (Supplemental Fig. 2b). Moreover, the numbers of total and unique specific reads were lower in the SGP than the IGP. On the basis of the degradome sequencing, 74.85% of the total reads (Supplemental Fig. 2c) and 14.30% of the unique reads (Supplemental Fig. 2d) overlapped in the SGP and IGP.

**Table 2:** Classification of annotation sRNAs of two small RNA and degradome libraries.

	Category	Superior		Inferior	
		Unique sRNAs	Total sRNAs	Unique sRNAs	Total sRNAs
Small RNA data	Total	6507147 (100%)	24071134 (100%)	8199346 (100%)	25301033 (100%)
	miRNA	52027 (0.8%)	966251 (4.01%)	57192 (0.7%)	971855 (3.84%)
	rRNA	96747 (1.49%)	1501425 (6.24%)	97844 (1.19%)	1679044 (6.64%)
	snRNA	4616 (0.07%)	30362 (0.13%)	4207 (0.05%)	31528 (0.12%)
	snoRNA	4915 (0.08%)	34710 (0.14%)	3841 (0.05%)	31194 (0.12%)
	tRNA	12285 (0.19%)	568690 (2.36%)	11633 (0.14%)	517882 (2.05%)
	unann	6336557 (97.38%)	20969696 (87.12%)	8024629 (97.87%)	22069530 (87.23%)
Degradome data	Total	4081576 (100%)	19169019 (100%)	8047259 (100%)	25468373 (100%)
	rRNA	27762 (0.68%)	7951866 (41.48%)	40475 (0.50%)	2188412 (8.59%)
	tRNA	1385 (0.03%)	3062 (0.02%)	2780 (0.03%)	6306 (0.02%)
	snRNA	2247 (0.06%)	27769 (0.14%)	4608 (0.06%)	78260 (0.31%)
	snoRNA	2949 (0.07%)	50768 (0.26%)	5315 (0.07%)	137062 (0.54%)
	polyN	2332 (0.06%)	3563 (0.02%)	6156 (0.08%)	9957 (0.04%)
	cDNA_sense	1866870 (45.74%)	4982151 (25.99%)	3725496 (46.30%)	10666028 (41.88%)
	cDNA_antisense	769380 (18.85%)	2222555 (11.59%)	1519460 (18.88%)	4696461 (18.44%)
	other	1408651 (34.51%)	3927285 (20.49%)	2742969 (34.09%)	7685887 (30.18%)

### Identification of known miRNAs in superior and inferior grains

**Figure 2:**
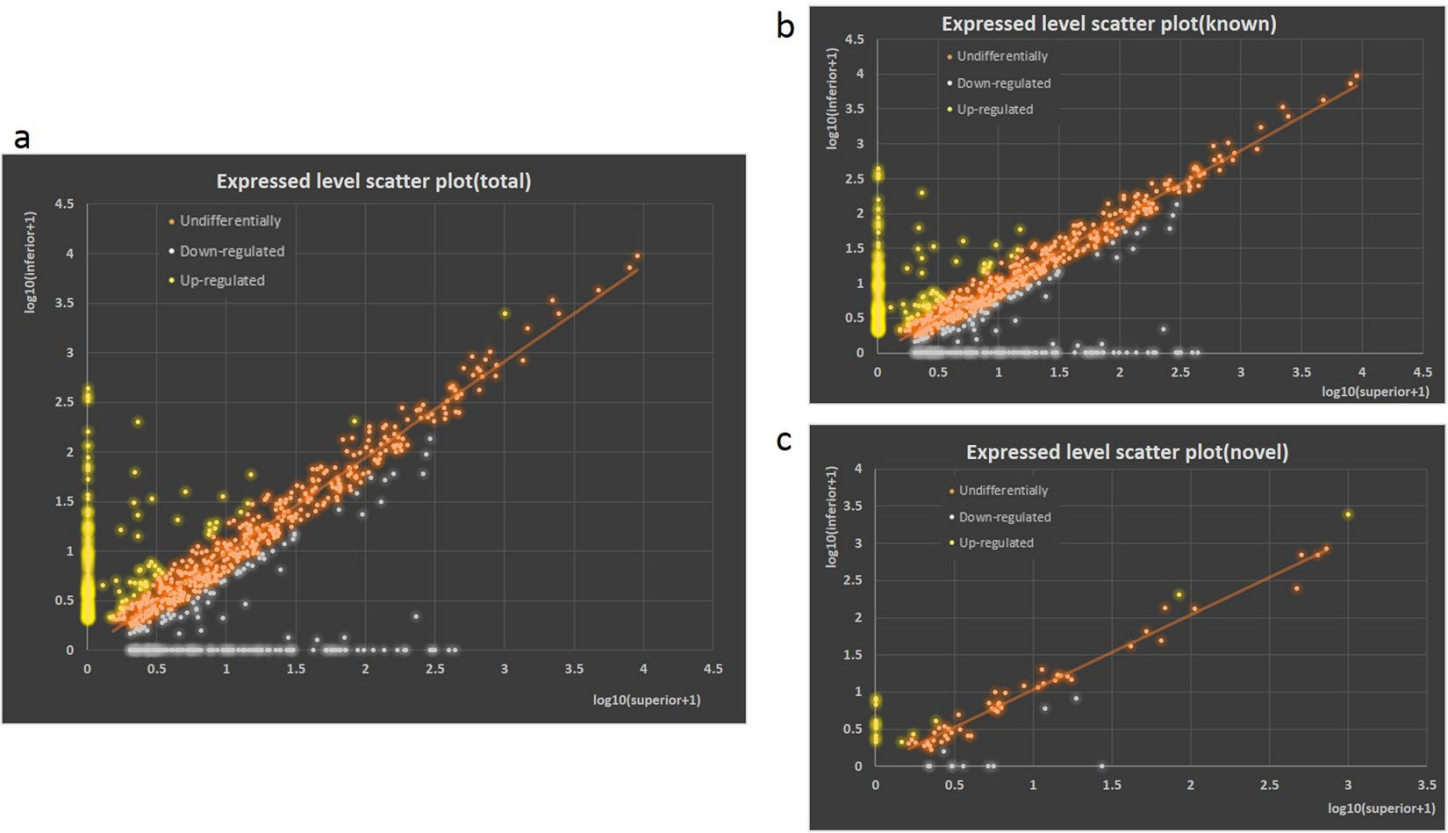
Scatter plots of conserved and novel miRNAs from SGP and IGP. X-axis was the miRNAs expressed abundance in SGP, and Y-axis was the miRNAs expressed abundance in IGP.

**Figure 3:**
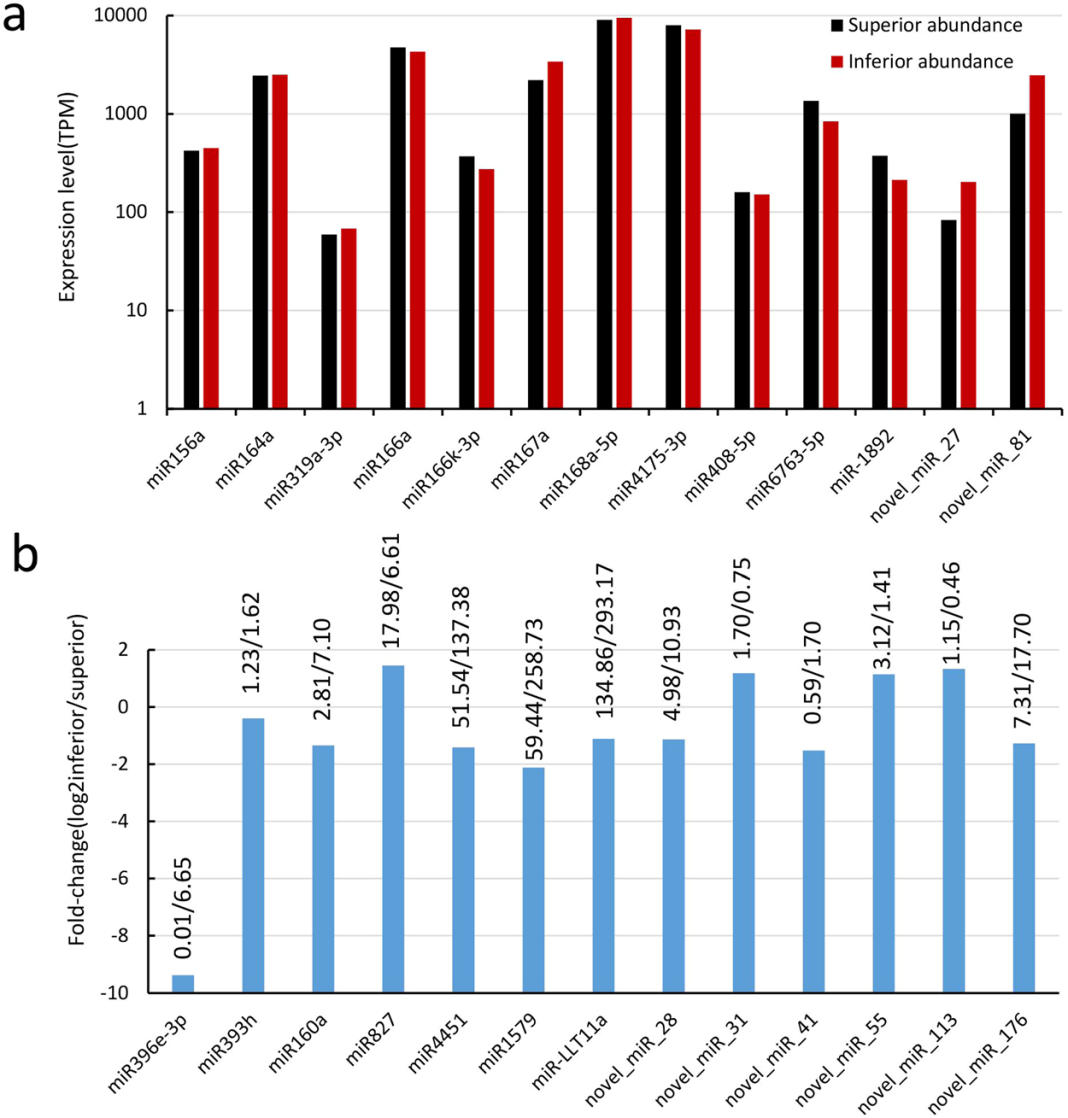
Selected conserved and novel miRNAs and their expression and fold-change in SGP and IGP. (**a**) The miRNAs with high expressed abundance. (**b**) The miRNAs with significantly difference in SGP and IGP.

To identify the conserved miRNAs of wheat, the small RNA clean reads of the libraries were searched against the known miRNA precursors and mature miRNAs from the miRBase^21,22^. Overall, we detected 746 miRNAs from small RNA sequencing (Fig. 2b), while we obtained 620 and 623 known miRNAs by the small sequencing method from the SGP and IGP, respectively. However, we obtained 497 miRNAs from both SGP and IGP, with 306 miRNAs up-regulated and 191 miRNAs down-regulated. Interestingly, differential expression analysis revealed that 97 known miRNAs were extremely significantly differentially expressed between the SGP and IGP libraries. Moreover, five (miR167a, miR164a, miR166a, miR4175-3p, and miR168a-5p) of the 306 miRNAs showed a relatively high expression level above 2000 TPM (transcripts per million) (Fig. 3a) and played roles in cell enlargement, nutrient metabolism, and stress resistance during grain development.

### Characterization of novel miRNAs in the libraries of superior and inferior seeds

Identification of novel miRNAs could broaden the miRNA database and enable a better understanding of the genetic basis of traits in view of epigenetics in bread wheat. All of the remaining unannotated sRNAs were predicted to be potentially novel miRNAs^21^. Based on their hairpin structures and specificity of sequence, we predicted 74 novel miRNAs in the SGP and IGP, 31 of which were up-regulated and 43 that were down-regulated (Fig. 2c). Additionally, we predicted 56 novel miRNAs in both the SGP and IGP. Analysis of the miRNA first nucleotide bias and miRNA nucleotide bias in each position demonstrated that the 10th and 11th nucleotide in most of the miRNAs, which matched the cleavage site of targets, were usually adenine (A) (Supplemental Fig. 3b,d). Moreover, U was the most frequent nucleotide at positions 20 and 22 in these miRNAs (Supplemental Fig. 3a,c).

Additionally, eight novel miRNAs (miR-27, miR-28, miR-31, miR-41, miR-55, miR-81, miR-113, and miR-176) also revealed significantly differential expression between SGP and IGP (*P* < 0.01) (Fig. 3a,b). Of these, miR-81 showing more than 1000 TPM had the highest abundance among novel miRNAs (Fig. 3a). Interestingly, there were five novel miRNAs (miR-27, miR-31, miR-55, miR-81, and miR-113) showing significantly higher differential abundance in IGP than in SGP. Thus, these predicted novel miRNAs would better broaden the miRNA database of wheat.

### Degradome analyses and target prediction

**Figure 4:**
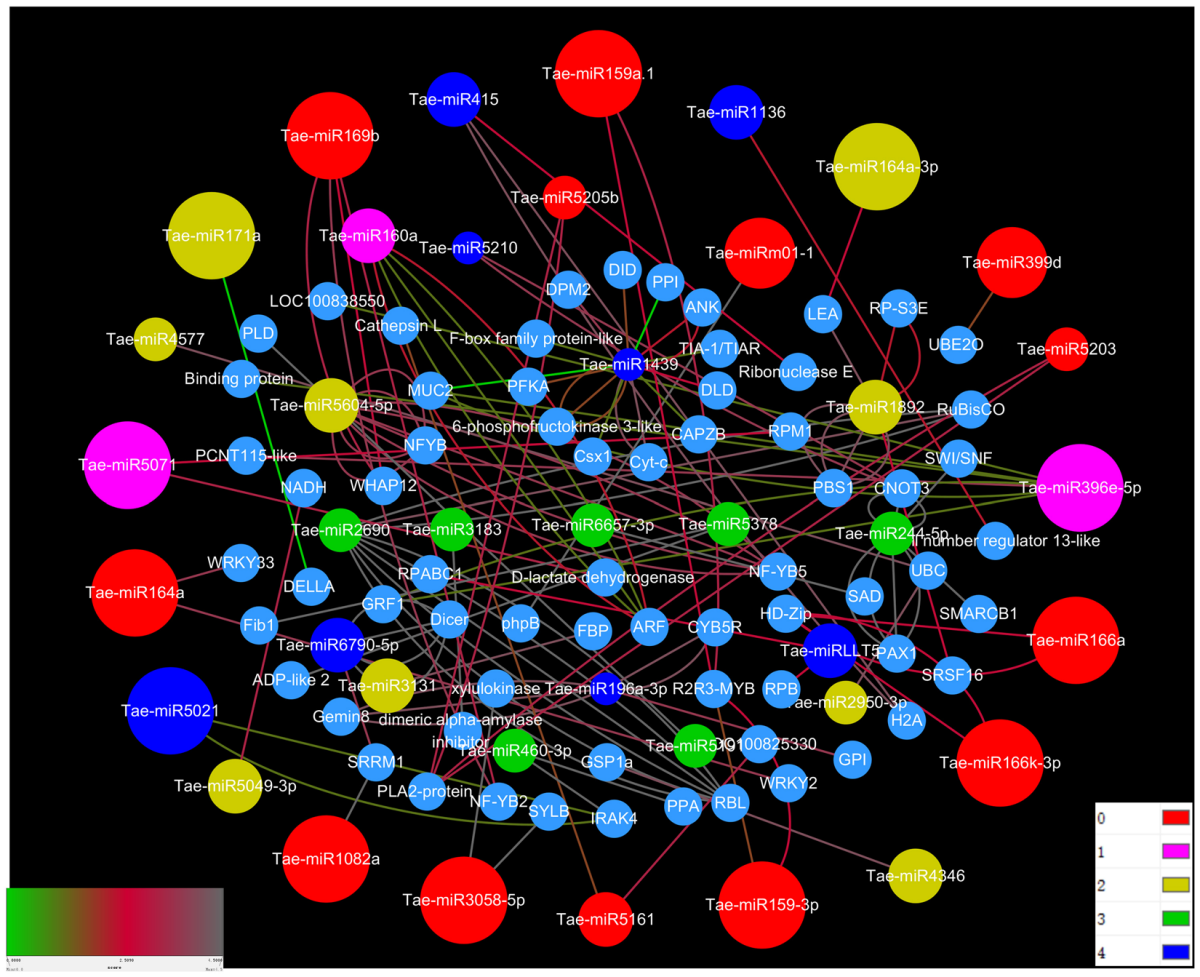
Relationships between miRNAs and their predicted targets in SGP and IGP showing a complex network visualized via the Cytoscape platform.

**Figure 5:**
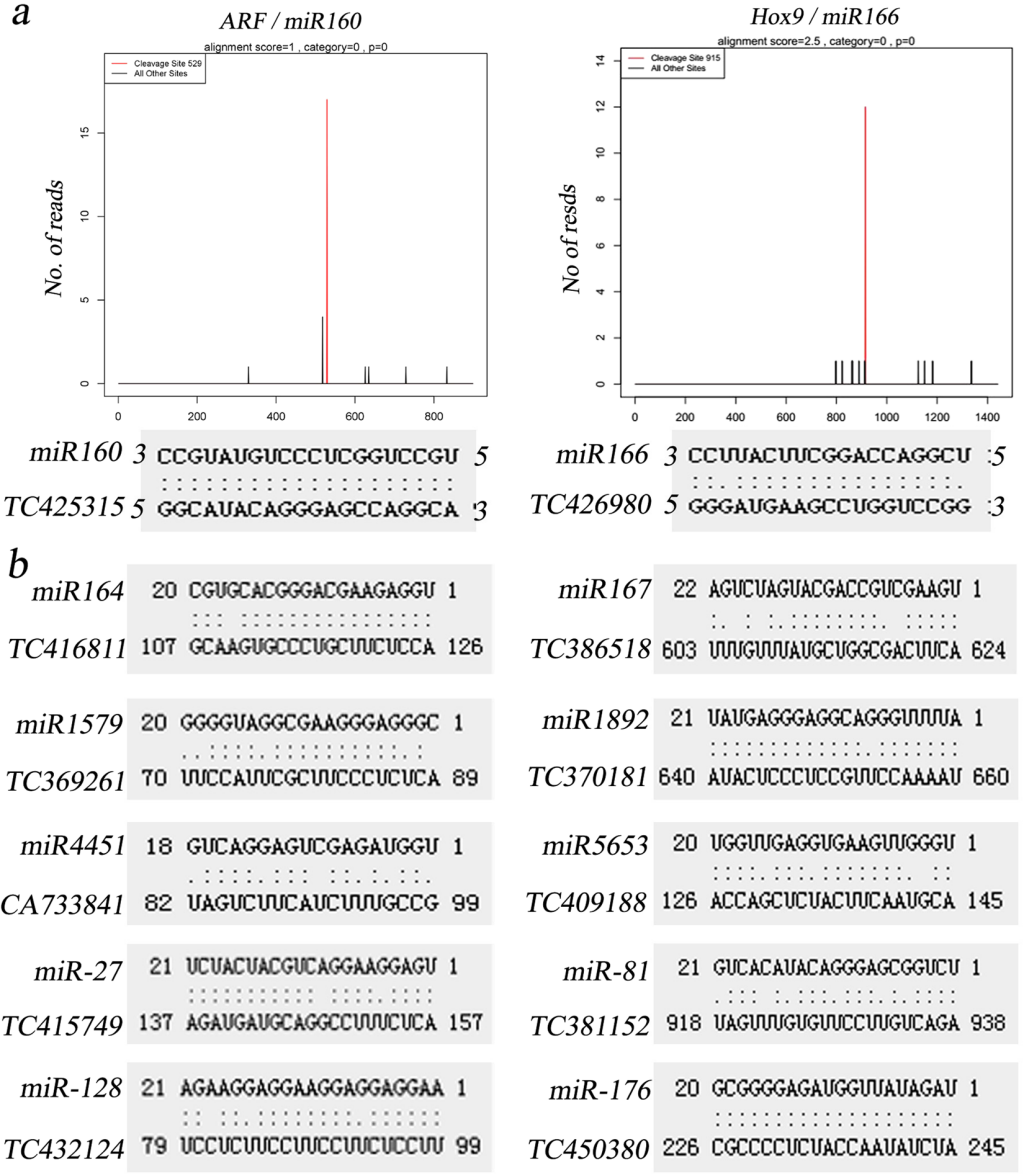
Verified targets of conserved development-related miRNAs. (**a**) Confirmed of target mRNA cleavage by degradome sequencing. The red lines indicated sequence reads consistent with miRNA-directed cleavage. (**b**) Mapping of target mRNA and miRNA alignment using psRNATarget. Accession numbers obtained from DFCI Wheat Gene Index (TAGI) (ftp://occams.dfci.harvard.edu/pub/bio/tgi/data/Triticum_aestivum/TAGI.release_12.zip) (TC or CA) are shown.

In this study, we used degradome sequencing and the psRNATarget server to predict miRNA target transcripts. In degradome sequencing, the results indicated that a total of 142 cleavage sites were gained to be associated with 39 miRNA families between superior and inferior grains. According to the totals of target genes, a network was constructed to show the interaction of miRNAs and their target genes using the Cytoscape platform (Fig. 4, Supplemental Table S3). These cleavage sites were identified with degradome peaks that were classified into five categories (0, 1, 2, 3, and 4) according to the peak height at the miRNA position, with targets of category 0 or 1 considered to be the most significant class. Degradome sequencing revealed that 10 miRNA families, including tae-miR159/*MYB*, tae-miR160/*ARF*(auxin response transcription factors), tae-miR164/*LEA* (late embryogenesis abundant), tae-miR166/*Hox9* (Homeodomain leucine-zipper protein) and tae-miR396/GRF(growth-regulating factor), possessed 29 target genes with multifunctional proteins in category 0. Interestingly, cleaved genes were not detected in category 1 between SGP and IGP, but tae-miR5071 was identified in SGP. Moreover, tae-miR399d and tae-miRm01-1 appeared in IGP at category 1. Additionally, we identified 25 target genes associated with embryo development and stress resistance protein *LEA* and *NF-YB* gene family members such as tae-miR1892, tae-miR5604, tae-miR3131, tae-miR5049, and tae-miR4346 in category 2. In the remaining categories (3 and 4), 42 target genes were detected. Importantly, we verified targets of conserved development-related miRNAs by degradome sequencing, such as miR160/*ARF* and miR166/*Hox9* (Fig. 5a).

In psRNATarget, we predicted miRNAs composed of 497 conserved miRNAs and 74 novel miRNAs to have 23,969 targets (Fig. 5, Supplemental Table S4), including transcription factors, ubiquitin-mediated proteolysis, signal transduction, and phytohormone signaling, such as *MYB*/miR159, *WRKY*/miR164, *NAC*-domain/miR164, *ARF*/miR160, *LEA* protein/miR1892, and *GRF*/miR396.

**Figure 6:**
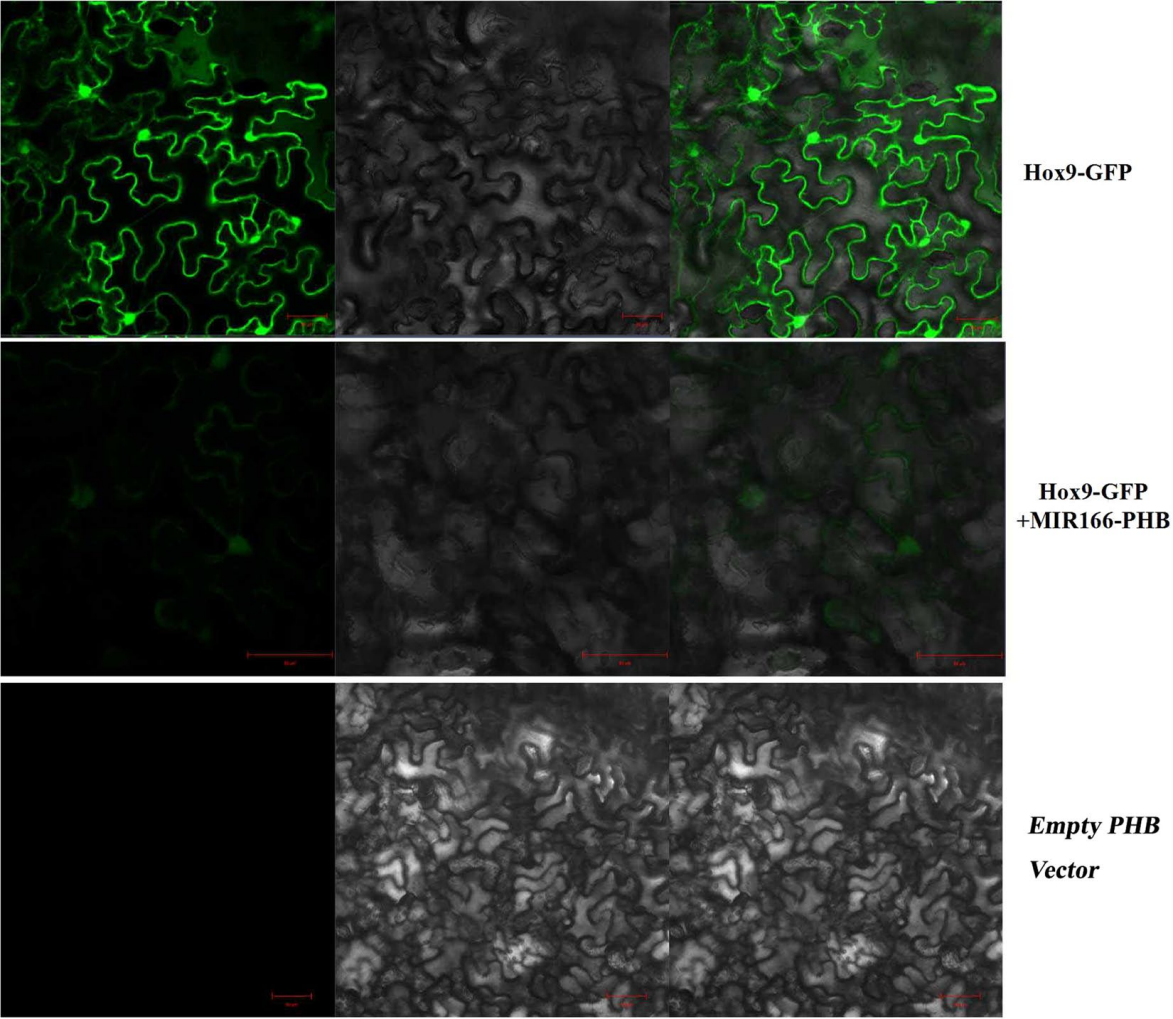
Transient expression assay for the regulation of miR166 to Hox9 observed by laser confocal microscope. Scale bar: 50 μm.

Transient expression system results showed that transgenic tobacco epidermal with empty *PHB* vector did not show any fluorescent signal, and transgenic tobacco epidermal with *Hox9-GFP* subcellular localization vector showed strong fluorescence signal in the cell membrane and nucleus. Transgenic tobacco epidermal with co-conversion *Hox9-GFP* and miR166*-PHB* vectors showed weak fluorescence signals (Fig. 6). Therefore, the results showed that miR166 inhibited *Hox9* gene expression.

### Validation of expression levels of miRNAs and their target genes in superior and inferior grains by qRT-PCR

**Figure 7:**
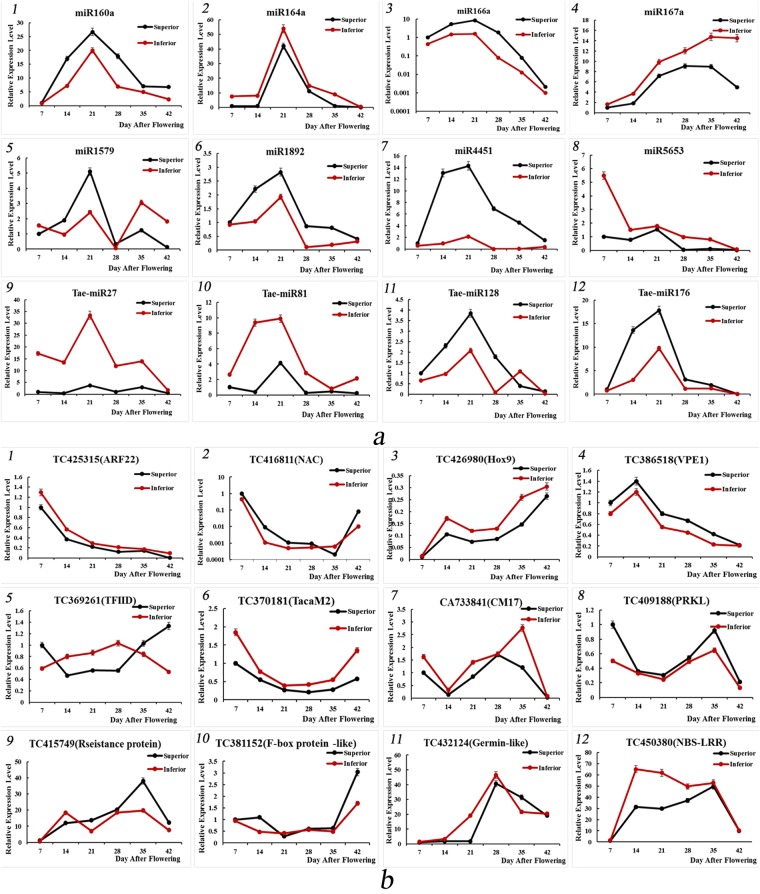
qRT-PCR validation of the 12 selected grain development-related miRNAs (**a**) and their targets (**b**) to measure their expression in SGP and IGP. No. of 1–12 are miR160a/TC425315 (Auxin Response Factor, *ARF*), miR164a/TC416811 (*NAC* transcription factor), miR166a/TC426980 (Homeodomain leucine-zipper protein, *Hox9*), miR167a/TC386518 (Vacuolar Processing Enzyme, *VPE1*), miR1579/C369161 (TATA box-binding protein, *TFIID*), miR1892/TC370181 (Calmodulin *TacaM2*), miR4451/CA733841 (Chloroform and Methanol, *CM17*), miR5653/TC409188 (kinase R-like protein, *PRKL*), novel-miR27/TC415749 (Resistance protein), novel-miR81/TC381152 (F-box protein-like), novel-miR128/TC432124 (Germin-like protein precursor) and novel-miR176/TC385289 (*NBS-LRR* resistance-like protein).

A total of 97 known miRNAs and 8 novel miRNAs were significantly differentially expressed between SGP and IGP based on fold change (≥1 or ≤−1) and P-value (<0.01) criteria. To validate the abundance of high-throughput sequencing, we conducted quantitative real-time PCR (qRT-PCR) to identify the expression patterns of these miRNAs (Fig. 7a). Based on the abundance and predicted functions of these miRNAs or their targets, we selected eight known miRNAs (miR160a, miR166a, miR1579, miR1892, and miR4451 showing up-regulated expression, and miR164a, miR167a, and miR5653 showing down-regulated expression) and four novel miRNAs (novel-miR128 and novel-miR176 showing up-regulated expression, and novel-miR27 and novel-miR81 showing down-regulated expression) to identify their expression levels in differently developed seeds of AK58 by qRT-PCR. The expression levels of miR160a, miR166a, miR1579, miR1892, miR4451, novel-miR128, and novel-miR176 were significantly up-regulated from 7 DAF to 21 DAF, with a peak at 21 DAF in SGP. Conversely, the expression levels of miR160a, miR166a, miR1892, miR4451, and novel-miR176 were decreased from 28 DAF to 42 DAF in SGP. Taken together, these results indicated that the expression of seven up-regulated miRNAs was higher in SGP than in IGP, and that they peaked at 21 DAF during the grain-filling stage. The miR164a, miR167a, miR5653, novel-miR27, and novel-miR81 showed down-regulated expression and lower expression in SGP. In addition, miR164a, novel-miR27, and novel-miR81 reached the highest expression at 21 DAF in IGP. However, the expression of miR167a increased from 7 DAF to 42 DAF, while the expression of miR5653 decreased during the grain-filling stage in IGP.

To identify the effects of miRNAs on their targets during the grain-filling stage, expression profiles of the predicted targets of 12 development-related miRNAs by the psRNATarget (Fig. 5b) were also validated by qRT-PCR (Fig. 7b). The results indicated that all targets containing TC425315 (*ARF*), TC416811 (*NAC*), TC426980 (*Hox9*), TC386518 (*Vacuolar Processing Enzyme, VPE1*), TC369161 (*TATA box-binding protein, TFIID*), TC370181 (*Calmodulin TacaM2*), CA733841 (*Chloroform and Methanol, CM17*), TC409188 (*kinase R-like protein, PRKL*), TC415749 (Resistance protein), TC381152 (F-box protein-like), TC432124 (Germin-like protein precursor), and TC385289 (*NBS-LRR* resistance-like protein) showed converse expression profiles when compared with their corresponding miRNAs, including miR160a, miR164a, miR166a, miR167a, miR1579, miR1892, miR4451, miR5653, novel-miR27, novel-miR81, novel-miR128, and novel-miR176 expression levels during the same grain-filling periods of superior and inferior grains. Interestingly, miR160a and miR166a peaked at 21 DAF, but the expression of *ARF* fell from 7 DAF to 42 DAF, whereas expression of the *Hox9* gene increased during the grain-filling period in IGP. These findings suggest that the miRNAs and their targets may have had a negative relationship in the specific tissues and grain development stages.

### GO enrichment and KEGG pathway analyses

**Figure 8:**
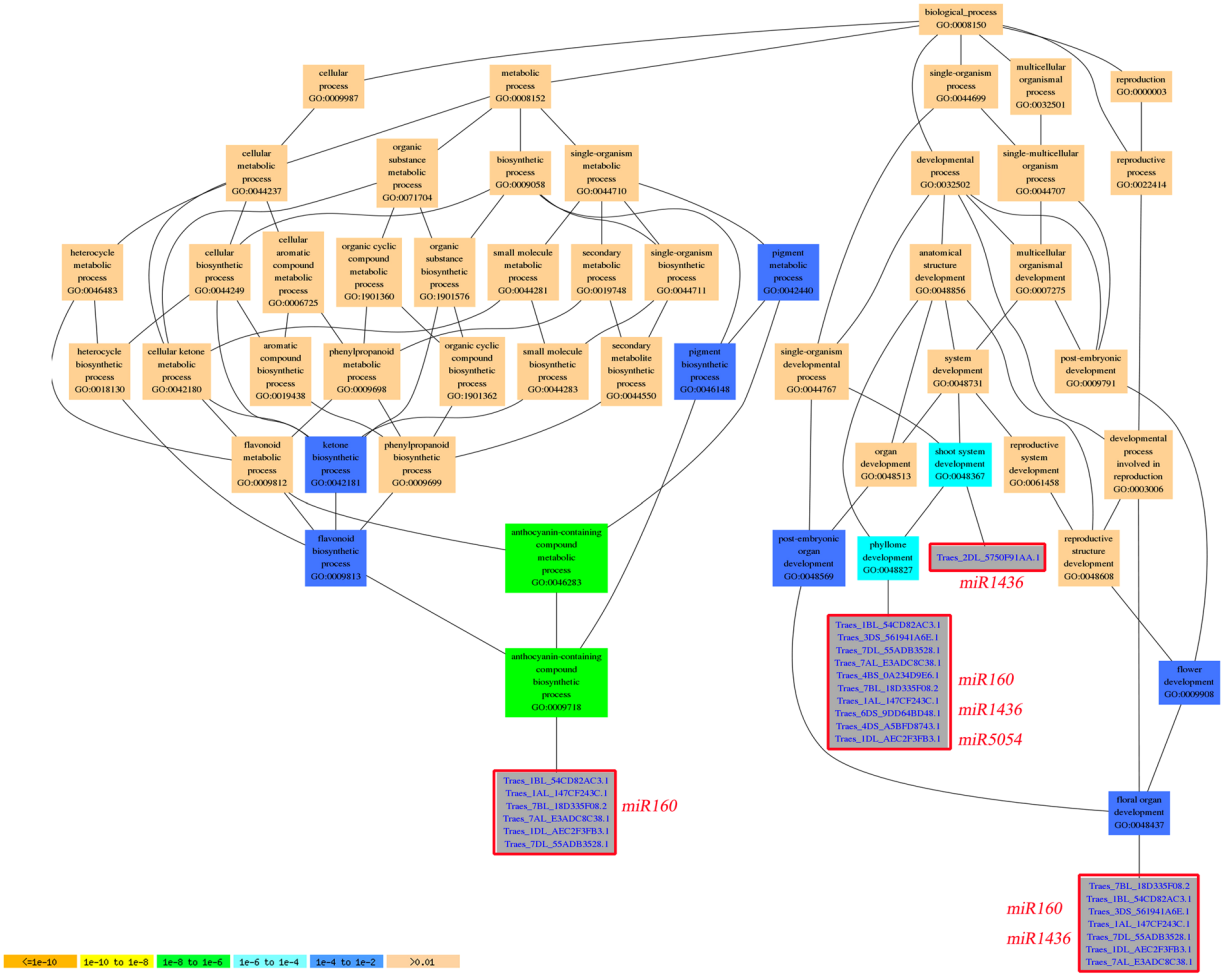
Diagrams of enriched GO biological process of grain development-associated miRNA targets constructed by AGRIGO. Different colors represented different significance levels.

To better identify roles of miRNAs in wheat grain development, we used Gene Ontology (GO)-based enrichment analysis to investigate the miRNAs that differed significantly between SGP and IGP. Our results indicated that a total of 1261 and 3309 mRNAs were targeted by known miRNAs and novel miRNAs. The targets of conserved miRNAs were involved in 17 biological pathways, 4 molecular functions, and 10 cellular components, whereas novel miRNAs were involved in 20 biological processes, 7 molecular functions, and 10 cellular components (Supplemental Fig. 4). In addition, the hierarchical graphs of all GO terms were built and showed the network for biological processes to demonstrate the directly and indirectly relative with miRNAs and their targets (Fig. 8; Supplemental Tables S5 and S6). Cellular processes (GO: 0009987), metabolic processes (GO: 0008152), and single-organism processes (GO: 0044699) were the top three biological processes involved in some miRNAs and their targets. In addition, miR160 was focused on athocyanin-containing compound metabolic process (GO: 0046283). Moreover, miR1436 played a critical role in shoot system development (GO: 0048367), which is another process of phyllome development (GO: 0048827) involved in miR160, miR1436, and miR5054. Floral organ development (GO: 0048437), which was associated with miR160 and miR1436, played vital roles in grain development for cereal crops.

**Figure 9:**
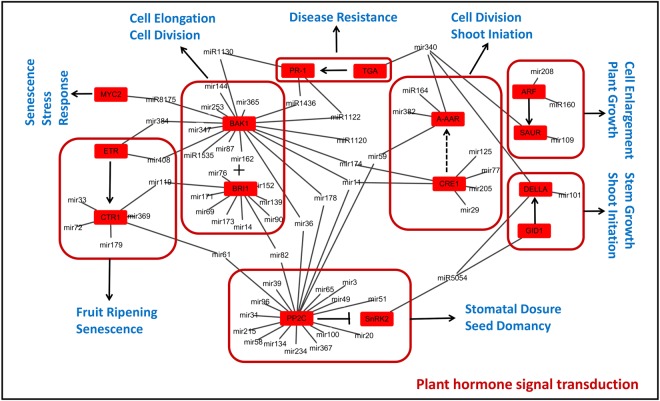
Known and novel miRNAs were involved in the plant hormone signal transduction pathway. Expression of these target genes differed significantly in the pathway.

The Kyoto Encyclopedia of Gene and Genome annotation indicated that we obtained 145 KEGG pathways, 23 of which were predicted to be related to grain development and filling (including metabolic pathways, plant-pathogen interactions, plant hormone signal transduction, starch and sucrose metabolism, nitrogen metabolism, fatty acid metabolism and so on). Further analysis, the plant hormone signal transduction pathway was associated with 15 target genes of 81 conserved and novel miRNAs (Fig. 9). Cell enlargement plant growth and cell division shoot initiation were involved in the tae-miR160 family and tae-miR164 regulating *ARF* and *A-ARR* genes. Moreover, ubiquitin mediated proteolysis and carotenoid biosynthesis were involved in the plant hormone signal transduction pathway, including *GID1, DELLA*, and *SnRK1* with one miRNA, tae-miR5054 regulating. The *BAK1* gene was regulated via tae-miR1436, tae-miR1535, tae-miR8175, tae-miR1120, tae-miR1122, and tae-miR1130, while *MYC2* was involved in stress response by tae-miR8175, and *PR-1* was regulated by tae-miR1436, tae-miR1122, and tae-miR1130 to affect the disease resistance pathway.

### Genetically modified validation in *Arabidopsis thaliana*

**Figure 10:**
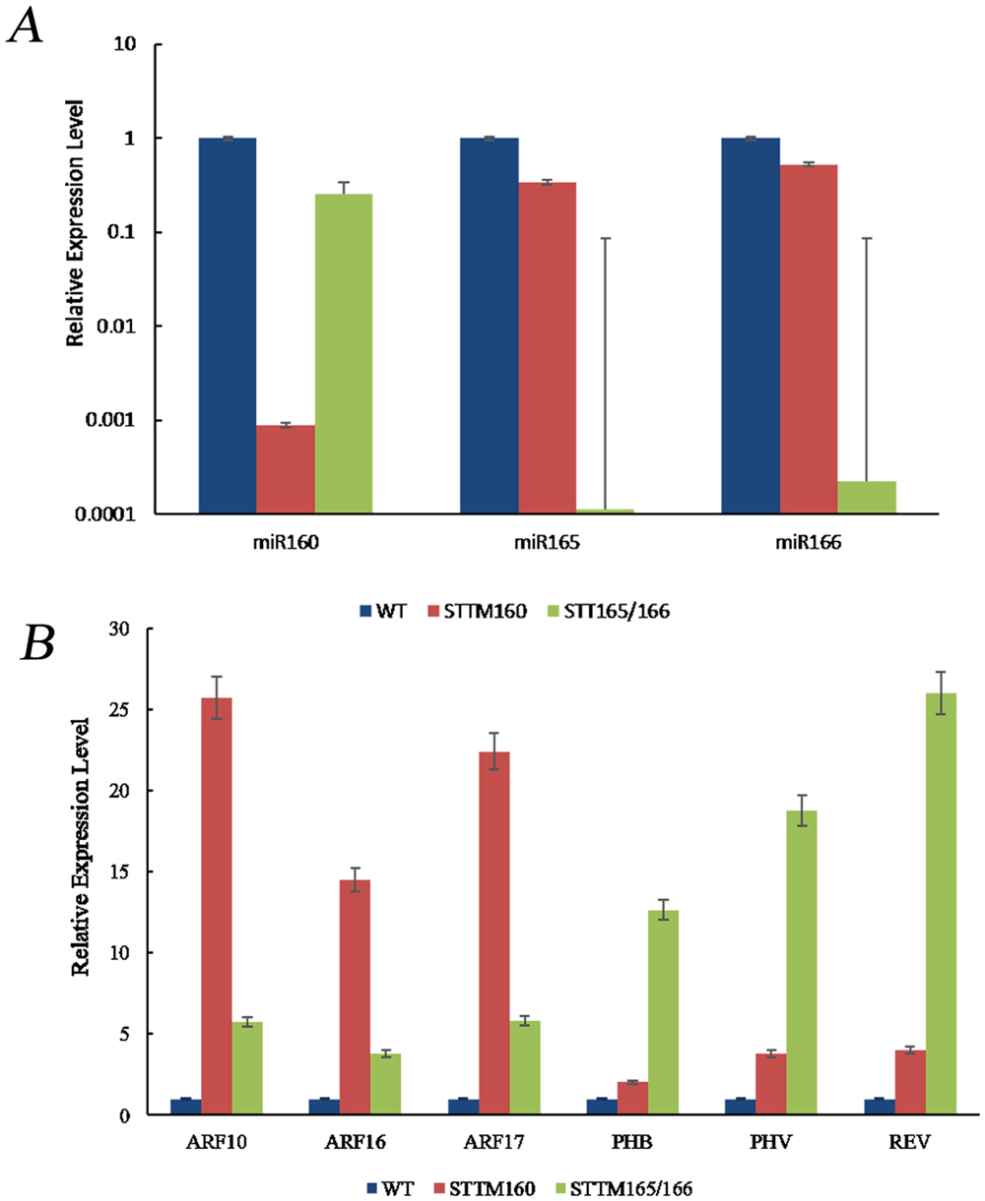
The miRNA/target expression patterns between WT and transgenic plants STTM160 and STTM165/166 using qRT-PCR. (**A**) qRT-PCR validation of miRNAs between WT and transgenic plants STTM160 and STTM165/166. (**B**) qRT-PCR validation of targets in WT and transgenic plants STTM160 and STTM165/166.

**Figure 11:**
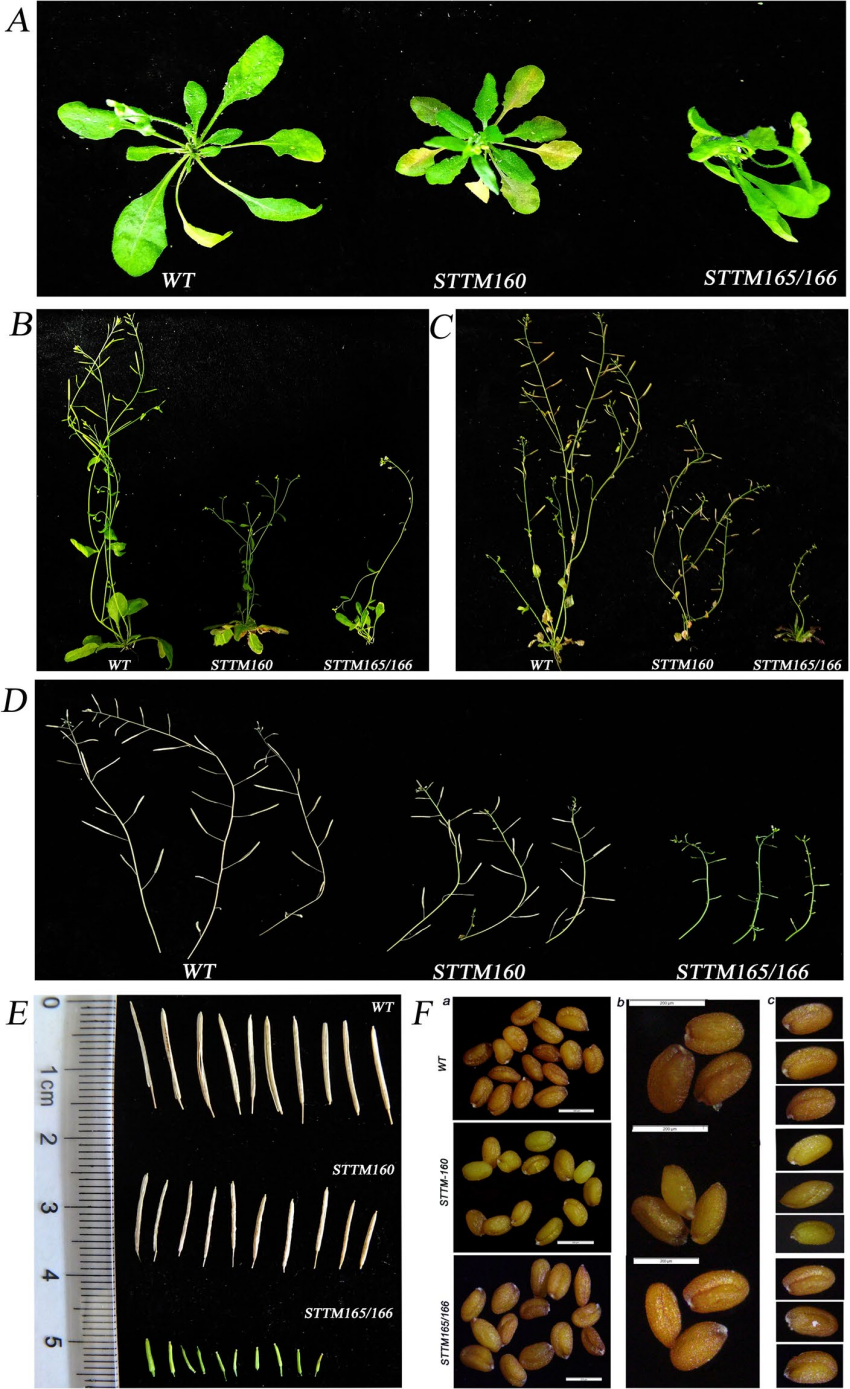
The phenotypes of Col-0, transgenic plants STTM160 and STTM165/166, and their seeds as observed by a microscope. (**A**) The Col-0, STTM160 and STTM165/166 plants at 30 days after germination (DAG). (**B**) The Col-0, STTM160 and STTM165/166 plants at 35 DAG. (**C**) The Col-0, STTM160 and STTM165/166 plants at 42 DAG. (**D**) The branches of Col-0, STTM160 and STTM165/166 selected for characterizing siliques and seeds. (**E**) Comparison of siliques of Col-0, STTM160 and STTM165/166. (**F**) The comparison of seeds from Col-0, STTM160 and STTM165/166 under the microscope.

According to a previous study^15^, the STTM-48nt transformants displayed obvious morphological phenotypes. Therefore, we utilized STTM technology to knock down miR160 and miR166, and identified the expression level of miR160 and miR165/166 and their corresponding targets (miR160/*ARF10, ARF16, ARF17* and miR165/166/*PHB* (PHABULOSA), *PHV* (PHAVOLUTA), *REV* (REVOLUTA)) by qRT-PCR in transgenic Arabidopsis (Fig. 10). When compared with wild-type Columbia-0 background (Col-0), the phenotypes of transgenic plants with STTM160/160 and STTM165/166 were significantly different, including grain length, grain width, grain weight, siliques development, and flowering time. The transgenic STTM160 plants showed serrated leaves, significantly shorter plant height and siliques, significantly reduced number of siliques and seeds, and a flowering time that occurred five days earlier than that of the WT (Col-0) (Fig. 11A–E, Table 3). Furthermore, transgenic STTM160 seeds showed significantly narrower widths per 10 kernels, shorter lengths per 10 kernels and lower weights per 200 kernels than WT (Fig. 11F and Table 3). These findings suggest that miR160 plays a key role in Arabidopsis seed development.

**Table 3:** The phenotype of transformational Arabidopsis by statistics.

Trait	Col-0	STTM160	STTM165/166
Seed Length (µm)	175.24 ± 8.24	166.93 ± 8.59*	178.37 ± 11.74
Seed Width (µm)	122.91 ± 4.02	99.26 ± 8.99**	117.86 ± 12.56
Seed weight (mg/200)	4.15 ± 0.04	2.90 ± 0.08**	4.09 ± 0.04*
Siliques Length (cm)	1.36 ± 0.08	1.03 ± 0.09**	0.44 ± 0.06**
Siliques number per plant	37.0 ± 3.74	28.5 ± 3.72**	10.3 ± 2.41**
Seed number per silique	39.1 ± 5.28	29.1 ± 1.92**	1.0 ± 1**
Flowering Time(d)	28 ± 2	23 ± 2	35 ± 2

The transgenic STTM165/166 plants showed serious dwarfism, trumpet-shaped or upward cupped curly leaves, delayed flowering time by one week, significantly shorter siliques, and significantly decreased number of siliques and seeds (Fig. 11A–E, Table 3) than WT plants. The transgenic STTM165/166 plants also displayed severe infertility (Fig. 11D,E, Table 3), with a silique length of only 0.44 cm, an average of 10.3 siliques per plant, and an average of 1 seed per silique (Table 3). These findings further resulted in a significantly reduced seed number and yield per plant. However, the transgenic STTM165/166 grain length and width did not differ significantly from those of the WT, although the weight per 200 kernels was significantly lower (0.06 mg) than that of the WT (Fig. 11F and Table 3). These findings suggest that miR165/166 plays a key role in the development of Arabidopsis plants and seeds.

## Discussion

Bread wheat (Triticum aestivum L.) is one of the world’s most important staple foods, and the improvement of grain yield has been a perennial topic^8^. Therefore, this study investigated miRNAs that related to the development of wheat grains to promote grain yield. To better explore whether miRNA is involved in grain development, we identified the grain phenotype of AK58 throughout the filling period (Fig. 1), and used high-throughput sequencing to excavate the differentiated miRNAs. Therefore, we found 12 miRNAs and their corresponding targets related to the development of seeds. Moreover, we employed STTM technology to knock down miR160 and miR165/166 in Arabidopsis, and further confirmed that these two miRNAs contribute to grain development (Fig. 11). Therefore, the results suggest that miRNAs play a key role in development of wheat grains and provide a new perspective for understanding the roles of miRNAs in superior and inferior grains of wheat.

### Grain development of bread wheat has numerous miRNAs

The development of high-throughput sequencing of miRNA has led to the identification of many miRNAs involved in various plant functions, such as miR159, miR160, miR164 and miR398, which are conserved among various plants. Furthermore, many miRNAs related to grain development have been identified in wheat, barley, maize and rice^6,14,20,29–31^. To date, however, no studies have focused on systematical identification or expression analysis of miRNAs in wheat superior and inferior grains using deep-sequencing approaches. Hexaploid wheat has three homoeologous A, B, and D genomes from related progenitor species and its genome size is very large (≈ 17.9 Gb, or more than 40 times larger than the rice genome)^32^. These findings suggested that numerous miRNAs were hidden in hexaploid wheat; therefore, it is important to further explore novel miRNA and its function in the development of wheat grain. Here, using high-throughput sequencing, we found that the expression of many miRNAs differed between superior and inferior grains of AK58 during grain development, and we identified a total of 497 known miRNAs and 56 novel miRNAs in both superior and inferior grains via small RNA sequencing. Degradome sequencing to identify the miRNA targets revealed the presence of 142 targets of 39 known miRNA family members in both SGP and IGP (Supplemental Table S4). Previous studies indicated that differently expressed miRNAs and targets were likely to be involved in many biological processes, such as those involving hormones, cell division, and signal transduction in rice^20^. Similarly, differently expressed miRNAs and targets were found to be involved in metabolic processes, cellular component organization, and biogenesis and signaling in the present study. Further analysis indicated that a large number of targets were involved in floral organ development, flower development, and flavonoid biosynthetic processes. Overall, combined small RNA analysis and degradome sequencing from different developmental stages should provide more insight into the network of miRNAs and their targets^33^.

### Several miRNAs and targets might play crucial roles in a broad range of wheat grain development

**Figure 12:**
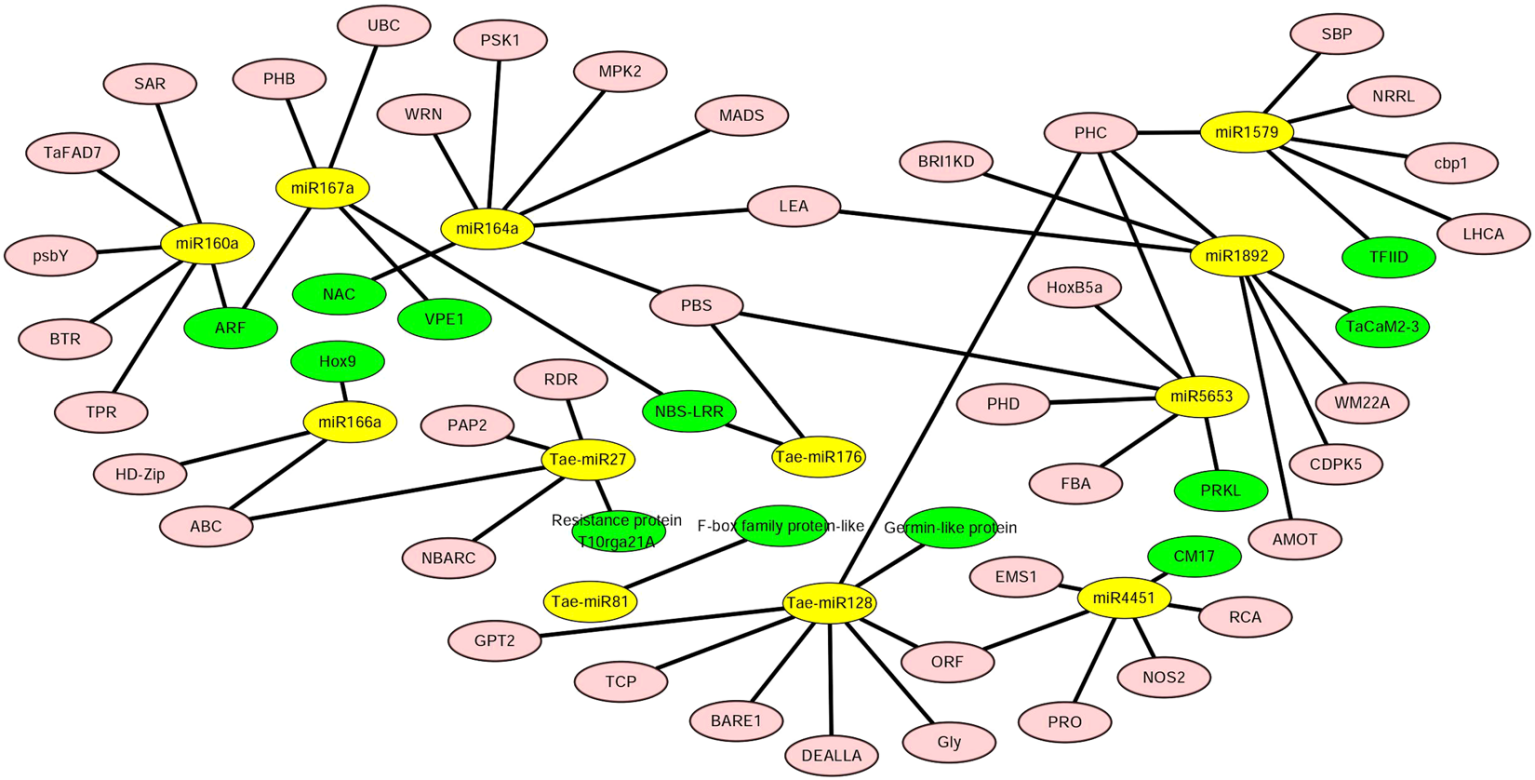
A network of miRNAs and their targets associated with grain development and validated by qRT-PCR. The yellow and green points represent validated miRNAs and targets, and pink points show other predicted targets that might play roles in grain development.

According to previously conducted studies, approximately 14 DAF was a major transition point as the start of grain filling and the end of cell division of the endosperm in wheat^34^. Another point occurred at about 28 DAF, when the grains decrease the deposition of storage and start to desiccate^6^. Therefore, we employed small RNA sequencing and degradome sequencing for profiling at 7, 14, 21, 28, 35, and 42 DAF to investigate the miRNAs related to superior and inferior grains. Based on our results, another interaction network explained the interrelationship of miRNAs and target genes that may have played a vital role in seed development (Fig. 12). For instance, miR160 played a key role in Arabidopsis, and its target gene *ARF* regulated plant growth and development. Moreover, miR160 exhibited a similar function in rice growth and development, including small seeds and dwarf stature^31^. In the present study, transgenic STTM160 plants showed significantly reduced seed width and silique length in Arabidopsis. These results indicated that miR160 potentially plays a key role in wheat grain to improve the inferior grain size. Jia *et al*. reported that miR165/166 showed the powerful functions in modulating auxin signaling and stress responses in Arabidopsis^16^, and displayed severe defects in the vegetative and reproductive stage. Members of the *HD-Zip* (*homeodomain-leucine zipper*) and *Hox9* were the target genes of miR166, and the *HD-Zip* has been shown to regulate leaf shape development in Arabidopsis^35^, while *Hox9* was found to play a role in wheat grain development^7^. Here, transgenic STTM165/166 plants showed significantly reduced seed number and sterile siliques in Arabidopsis, suggesting that miR166 plays vital roles in grain development and might be a useful evidence to improve inferior grain size in wheat. Several miRNAs have functions that have not been verified but that also may be related to the grain size development. For example, the targets of miR164, *NAC, PSK1* (*Phytosulfokinealpha 1*) and *PBS1* (*Chloroplast oxygenevolving enhancer protein 1*) influence cell proliferation in wheat grain development^7^. Moreover, *PHB*, *ARF*, *VPE1*, and *NBS-LRR* were shown to be the targets of miR167. Additionally, *NBS-LRR*, which is also regulated by miR160 and Tae-miR176, is a resistance-like protein that plays crucial roles in diverse communication processes, including cell proliferation, virus differentiation, and drought stress^36^. *VPE1* was found to be required for efficient glutelin processing in rice^37^, while *ARF* played a vital role in the IAA pathway in plant development^38^. As a member of the *HD-ZIP* family, the *PHB* gene was shown to mainly regulate leaf shape development and responses to abiotic stresses in Arabidopsis^16,35^. *LEA* gene, which is regulated by miR164 and miR1892, plays a key role in embryogenesis development and resistance to stress^39^. Furthermore, Tae-miR128 regulated *PHC* (*cell wall protein pherophorin*) and *TCP* (*cell division inhibitor-like*) genes, which are involved in cell wall development and cell division in plant growth^40^. In summary, most genes involved in stress and plant development were associated with miRNA in different plants. Therefore, miRNAs play vital regulatory roles during grain development and could be a powerful tool to improve grain size in wheat.

### Putative functional network of development-related miRNAs in wheat grains

**Figure 13:**
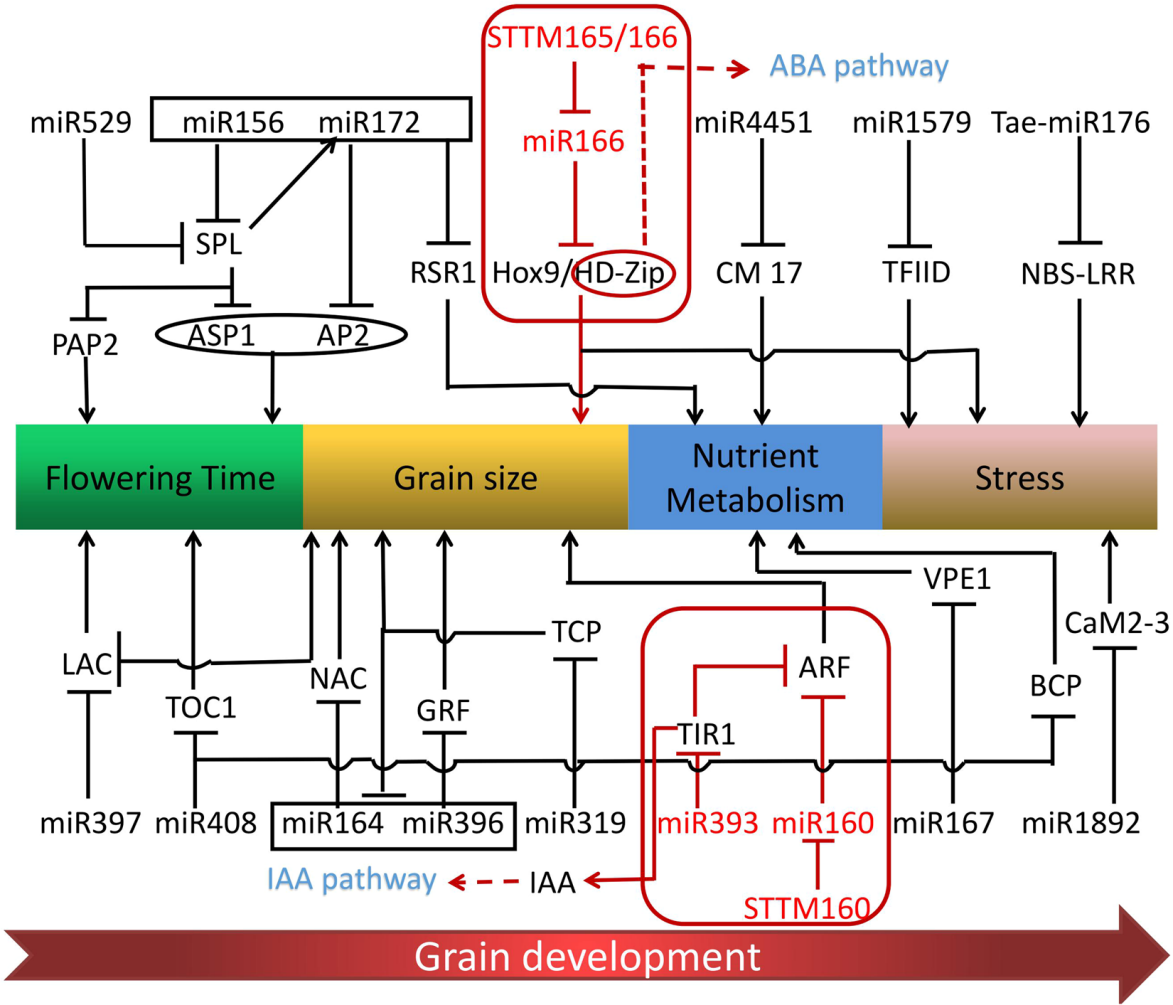
A model of grain development-related miRANs and their target genes involved in the regulation of grain growth and development. miRNAs and targets were related to several stages of grain development: flowering time, enlarged grain size, nutrients storage and the whole life-cycle of plant stressing. IAA and ABA pathway genes play a vital role in grain development.

Grain development as a unique biological process relies on the complex regulation of gene expression. Here, the exhibited network (Fig. 13) showed that miRNA played different roles in diverse grain development stages, such as flowering time, grain size, nutrient metabolism, and stress. For instance, some highly conserved miRNAs (e.g., miR156, miR172, miR529, and miR408) have been investigated for their role in flowering time in various crop species^13,41–43^. For instance, overexpression of tae-miR408 exhibited early heading phenotype in hexaploid wheat^42^. As the target of miR529 and miR156, *SPL14* transcription factor was encoded via a quantitative trait locus, *IPA1* (*ideal plant architecture 1*)^43^, which could enhance grain size and productivity in rice by promoting expression of a series of vital genes, including *OsTB1* (a protein that negatively regulates lateral Branching) and *OsGW8*^12,41,44^. Additionally, *SPL14* modulated the *PAP2* (*PANICLE PHYTOMER2*) gene to affect plant flowering time^41^; miR156 and miR172 were found to be related together to flowering time in Arabidopsis and maize^45^; and *SPL14, ASP1* (*ABERRANT SPIKELET AND PANICLE1*), and *AP2* (*APETALA2*) were shown to regulate floral organ development in rice^41^. As a target of miR172, *AP2* was found to play a key role in maize and barley^29,30,46^, and its presence led to significant floral defect and seed weight decrease. As a new potential target of miR156 and miR172, the starch negative regulator *RSR1* was predicted to play a crucial role in nutrient metabolism during the wheat grain filling stage.

In addition, miR160, miR166, miR164, miR319, miR393, miR396, miR397, and miR398 participated in modulation of grain size. As targets of miR160, *ARF* played a key role in the IAA pathway to modulate grain development, while the Hox9 gene regulated wheat grain development^7^. However, the expression of miR166 was inhibited via the *HD-Zip*, which influences cell division to regulate leaf shape and development in plants^47^. In addition, *HD-Zip* family up-regulates ABA content to affect the ABA pathway^16^. As a target of miR319, *TCP* transcription factors inhibited the expression of miR164 and miR396, whereas miR164 and miR396 were found to modulate activities of *NAC* and *GRF* and cell proliferation in wheat^7^. As a miRNA related to Auxin, miR393 contributes to rice seed development to target growth hormone receptor *TIR1* and the *AFB2* gene and also regulates the growth and development of tiller, leaf angle, and seed size^48^. *GRF*, the target of miR396, could promote expression of the *GS2* gene and thus lead to larger cells and increased numbers of cells promoting grain weight and yield through overexpression in rice^49^. OsmiR397 was naturally preferentially highly expressed in rice seeds^13^, and members of the miR397 family were present in diverse species to regulate grain size, grain number, and grain yield, including *Zea mays*, *Triticum aestivum*, and *A. thaliana*^50–52^). In addition, a reduced grain weight resulted from overexpressing miR398, which targets the *CSD* gene, leading to smaller seeds and reduced grain number per panicle in rice^53^.

Dry matter accumulation of wheat grains is vital to nutrient metabolism. Here, we found that three miRNAs (miR167, miR408, and miR4451) were associated with nutrient metabolism. As the target of miR408, *BCP* (*blue copper proteins*) might have participated in copper transportation and storage. *CM 17*, the target of miR4451, may modulate nutrient storage in cereal seeds^54^. Some miRNAs can greatly affect abiotic and biological stresses, such as drought and salinity tolerance. Here, we found three miRNAs (miR166, miR1892, and Tae-miR176) to be associated with stress. As a target of miR1892, *TaCaM2-3* (*Calmodulin*) may participate in plant signal transduction pathways^55^. *NBS-LRR*, the target of tae-miR176, could modulate disease resistance genes in the plant defense system of grain development^56^. Therefore, miRNAs are important noncoding genes that are involved in many biological processes of grain development and production. Overall, these miRNAs and their targets weaved a subtle functional network during grain development.

## Electronic supplementary material

Supplemental figure S1-4

Supplemantal Table S1-S6
