# Supplementary material for: High-throughput sequencing revealed that microRNAs were involved in the development of superior and inferior grains in bread wheat
Source: Sci Rep. 2018 Sep 14;8:13854. doi: 10.1038/s41598-018-31870-z (PMC6138641; doi:10.1038/s41598-018-31870-z)
Supplement: Supplementary file 1 — Supplemental figure S1-4 [file 41598_2018_31870_MOESM1_ESM.pdf]

Supplemental files

High-throughput sequencing revealed that microRNAs were involved in the development of superior and inferior grains in bread wheat

Wang Yongyan [1, 2†], Shi Chaonan[1†], Yang Tianxiao[1], Zhao Lei[1], Chen Jianhui[1], Zhang Ning[1], Ren Yan[1], Tang Guiliang[1, 2], Cui Dangqun[1], Chen Feng[1]*

[1] Agronomy College / National Key Laboratory of Wheat and Maize Crop Science / Collaborative Innovation Center of Henan Grain Crops, Henan Agricultural University, 15 Longzihu College District, Zhengzhou 450046, China

[2] Department of Biological Sciences, Life Science and Technology Instituted, Michigan Technological University, Houghton, MI 49931, USA

Y.W. and C.S. contributed equally to this paper.

Corresponding author: chf0088@163.com

Running title: miRNAs play keys role in seed development of wheat

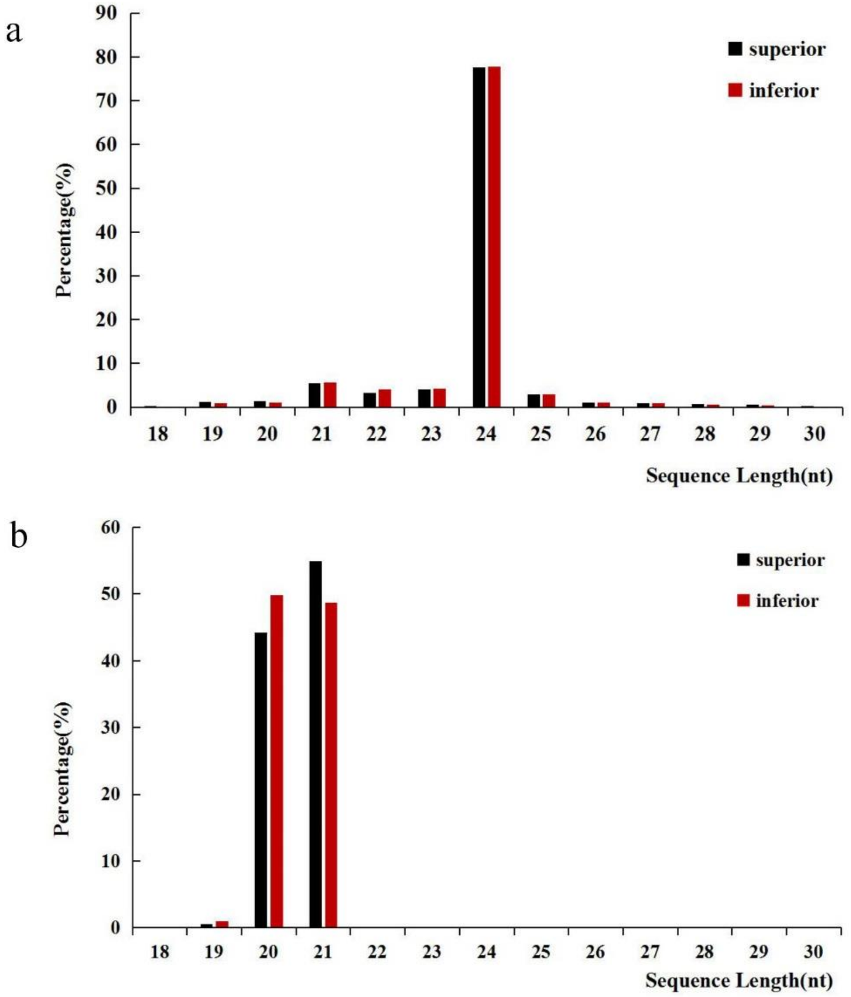

Figure S1 Small RNA sequence length distributions in superior and inferior grains by small RNA sequencing and degradome sequencing. (a) superior and inferior grains by small RNA sequencing, showing a major peak at 24 nt position and a minor peak at 21 nt position; (b) superior and inferior grains by degradome sequencing (DE), showing a major fractions of 20 and 21 nt sequences determined by DE.

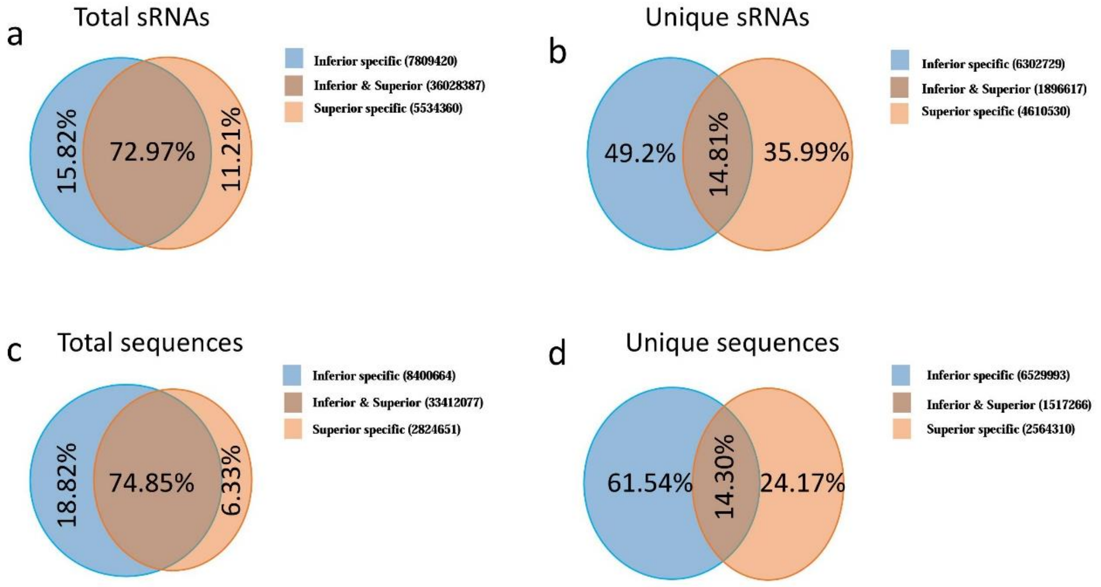

Figure S2 Analysis of total and specific sequences between superior grain pool (SGP) and inferior grain pool (IGP). (a) Summary of total sequences between SGP and IGP by small RNA sequencing. (b) Summary of unique sequences between SGP and IGP by small RNA sequencing. (c) Summary of total sequences between SGP and IGP by degradome sequencing. (d) Summary of unique sequences between SGP and IGP by degradome sequencing.

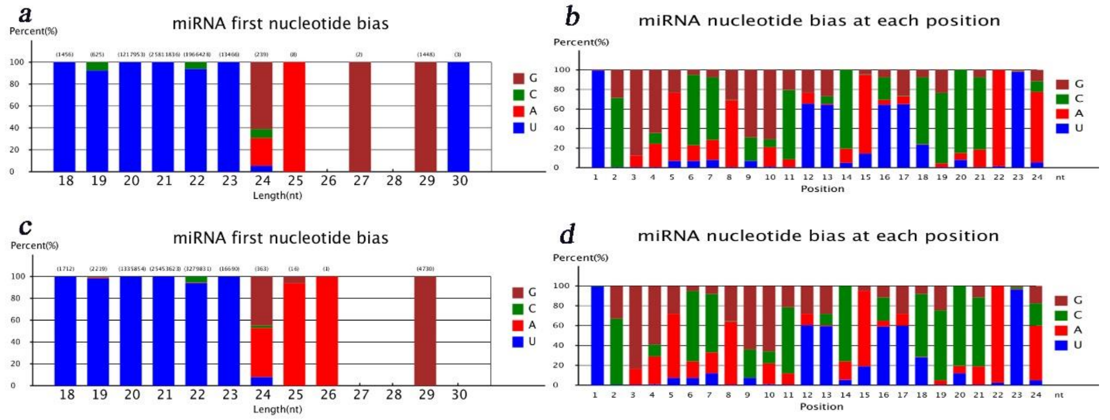

Figure S3 Distribution of miRNA nucleotide bias. (a): First nucleotide bias of 18~30nt novel miRNA candidates in SGP. (b): Nucleotide bias at each position of novel miRNA candidates in SGP. (c): First nucleotide bias of 18~30nt novel miRNA candidates in IGP. (d): Nucleotide bias at each position of novel miRNA candidates in IGP.

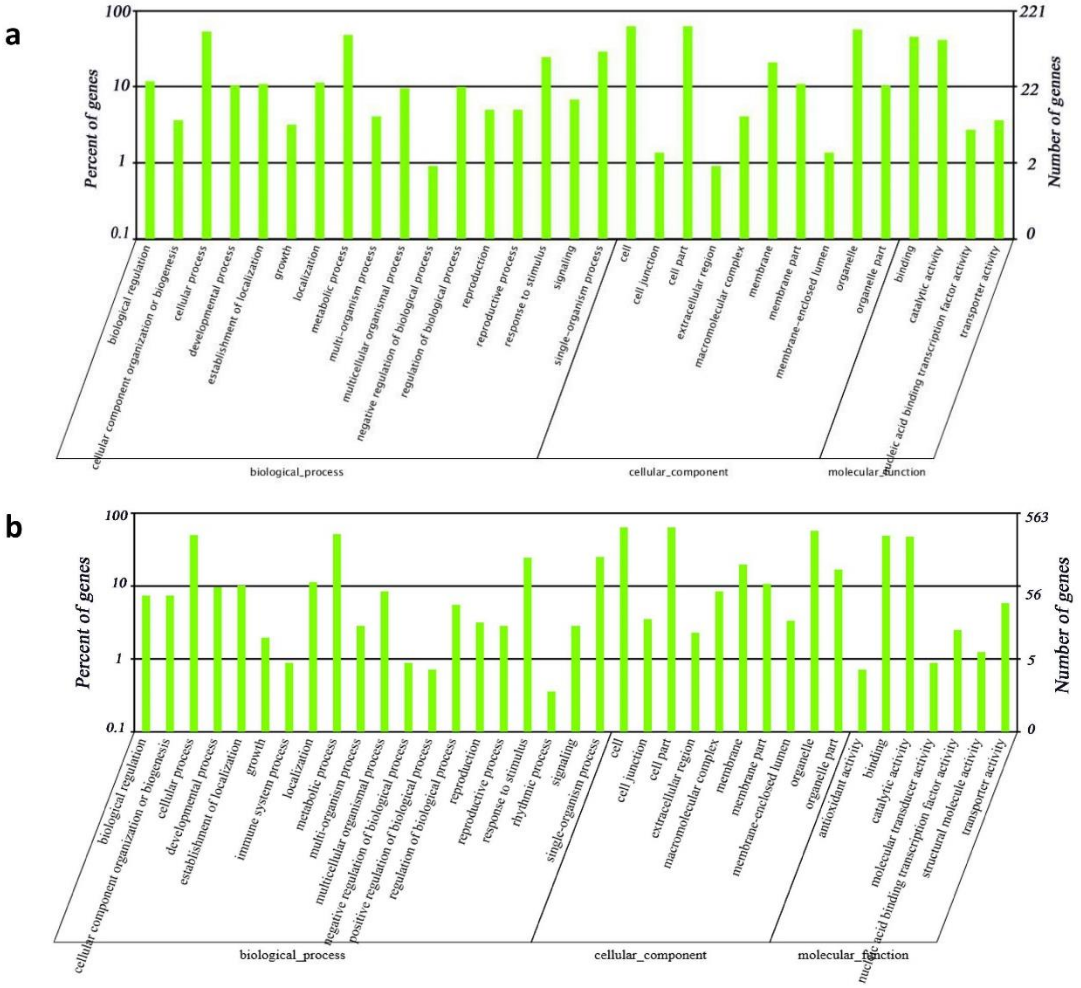

Figure S4 Functional annotation of GO analysis related to grain development for known and novel miRNA targets from SGP and IGP in AK58. (a) known miRNA targets; (b) novel miRNA targets.
